# Observational Comparative Study for Surgical Outcomes of One- or Two-Level Lumbar Fusion Surgery Between Transforaminal Lumbar Interbody Fusion and Lateral Lumbar Interbody Fusion

**DOI:** 10.3390/jcm15031066

**Published:** 2026-01-29

**Authors:** Seok-In Jang, Bong-Su Mun, Sang-Min Park, Ohsang Kwon, Jin S. Yeom, Ho-Joong Kim

**Affiliations:** Spine Center, Department of Orthopedic Surgery, Seoul National University College of Medicine, Seoul National University Bundang Hospital, Seongnam-Si 13620, Gyeonggi-Do, Republic of Korea; stanley007@naver.com (S.-I.J.); elitesu@naver.com (B.-S.M.); grotyx@gmail.com (S.-M.P.); ormssang@gmail.com (O.K.); highcervical@gmail.com (J.S.Y.)

**Keywords:** transforaminal lumbar interbody fusion, lateral lumbar interbody fusion, segmental lordotic angle, degenerative lumbar disease, patient-reported outcomes

## Abstract

**Background/Objectives:** Transforaminal lumbar interbody fusion (TLIF) and lateral lumbar interbody fusion (LLIF) are widely utilized techniques for degenerative lumbar diseases. This study compared radiological and clinical outcomes of LLIF and TLIF in patients undergoing lumbar fusion. **Methods:** This non-randomized prospective observational study enrolled 117 patients (LLIF: n = 17; TLIF: n = 100), with an inherent imbalance in group sizes, who underwent one- or two-level lumbar interbody fusion. Primary outcome was segmental lordotic angle (SLA) at the operated level. Secondary outcomes included disc height, lumbar lordotic angle, sagittal vertical axis, and patient-reported outcomes. Assessments were conducted at baseline, 3, 6, 12, and 24 months. Linear mixed models analyzed longitudinal data. **Results:** Mean SLA improvement was not significantly different between the groups (LLIF: 3.04° vs. TLIF: 3.18°, *p* = 0.782). No significant differences were observed for disc height (*p* = 0.518), lumbar lordotic angle (*p* = 0.718), or sagittal vertical axis (*p* = 0.866). Patient-reported outcomes improved significantly in both groups. Linear mixed model analysis revealed no significant between-group effects for Oswestry Disability Index (*p* = 0.335) or low back pain (*p* = 0.069). TLIF showed higher rates of dural tears and wound complications, while LLIF had more sympathetic chain injuries and transient psoas weakness. Overall complication rates were comparable (*p* > 0.05). **Conclusions:** TLIF and LLIF demonstrate comparable radiographic and clinical outcomes at 24-month follow-up. Surgical technique selection should be individualized based on patient-specific anatomical and clinical factors, considering distinct approach-specific complication patterns.

## 1. Introduction

Low back pain remains the leading cause of years lived with disability worldwide, with its prevalence and societal burden continuing to rise across regions as populations age [1]. Degenerative lumbar diseases, including lumbar spinal stenosis and degenerative spondylolisthesis, significantly impair patients’ quality of life by causing chronic back pain, radiculopathy, and functional limitations [2]. Surgical interventions, such as lumbar interbody fusion (LIF), have emerged as effective treatments for alleviating these symptoms and restoring spinal stability [3]. Among the various surgical techniques, transforaminal lumbar interbody fusion (TLIF) and lateral lumbar interbody fusion (LLIF) are widely utilized due to their distinct approaches and benefits [4]. TLIF employs a posterior approach, enabling direct visualization of neural elements and intervertebral disc spaces. Conversely, LLIF utilizes a lateral retroperitoneal approach, offering advantages such as reduced disruption of posterior spinal structures and greater ability to restore disc height and lumbar lordosis [5,6].

Restoration of sagittal alignment following LIF surgery has been recognized as a critical determinant of long-term outcomes. Recent studies have demonstrated a correlation between segmental lordotic angle (SLA) improvement and postoperative clinical outcomes. Wang et al. reported that among radiologic parameters, greater changes in SLA were significantly associated with achievement of the minimal clinically important difference (MCID) in patient-reported outcomes [7]. Kuhta et al. demonstrated that undercorrection of SLA following single-level TLIF negatively affected Oswestry Disability Index (ODI) scores up to 5 years postoperatively [8]. While both TLIF and LLIF have been shown to improve SLA, several investigations have suggested that LLIF may provide superior restoration compared to TLIF [9,10].

This study aims to compare the radiological and clinical outcomes of LLIF and TLIF in patients undergoing one- or two-level lumbar fusion surgery. The findings from this study are expected to inform surgical decision-making and optimize treatment strategies for patients with degenerative lumbar disease.

## 2. Methods

### 2.1. Study Design

This study is a non-randomized prospective observational comparative analysis conducted to evaluate the clinical and radiological outcomes of two lumbar fusion techniques: TLIF and LLIF. Patients undergoing one- or two-level lumbar interbody fusion for degenerative lumbar disease at a single institution were included in the study, between August 2020 and August 2023.

Patients were assigned to either the TLIF or LLIF group based on the preoperative surgical plan determined by the treating surgeon. The assignment was not randomized, as this study was designed as a single-centre, prospective study. The choice of surgical technique was influenced by patient-specific factors, including anatomical considerations, surgeon preference, and clinical indications. A priori sample size calculation, based on an expected SLA difference of 3.2° (SD 6.5°), alpha of 0.05, power of 80%, and 10% attrition, indicated 81 patients per group would be required. We targeted 100 patients per group for this observational study to provide adequate statistical power. However, challenges in enrolling suitable candidates for LLIF, along with factors such as device availability and study duration, led to an unequal final sample distribution. At the surgical level, if a patient had previously undergone discectomy or laminectomy, or if foraminal stenosis was identified as the main lesion rather than central stenosis, LLIF was favoured over TLIF. Conversely, in cases with severe central stenosis requiring direct decompression, pronounced facet arthrosis that made indirect decompression challenging, or a history of abdominal surgery, TLIF was the preferred approach.

### 2.2. Participants

Consecutive patients scheduled for one- or two-level lumbar interbody fusion for degenerative lumbar disease were screened for eligibility. Inclusion criteria comprised: (1) indication for lumbar interbody fusion via anterior or posterior approach at one or two contiguous levels; (2) clinical presentation of unilateral or bilateral lumbar radiculopathy or intermittent neurogenic claudication attributable to degenerative lumbar pathology; (3) age ≥ 18 years; (4) capacity to comply with the study protocol and follow-up schedule; and (5) provision of written informed consent.

Exclusion criteria included: (1) prior radiotherapy at the intended surgical level; (2) progressive motor deficit or cauda equina syndrome requiring emergent intervention; (3) active systemic or local infection; (4) skeletal immaturity; (5) active malignancy; (6) pregnancy; (7) symptomatic osteoporosis; (8) contraindication to general anesthesia; and (9) inadequate comprehension of the Korean language precluding valid questionnaire completion.

### 2.3. Surgical Procedures

**Figure 1:**
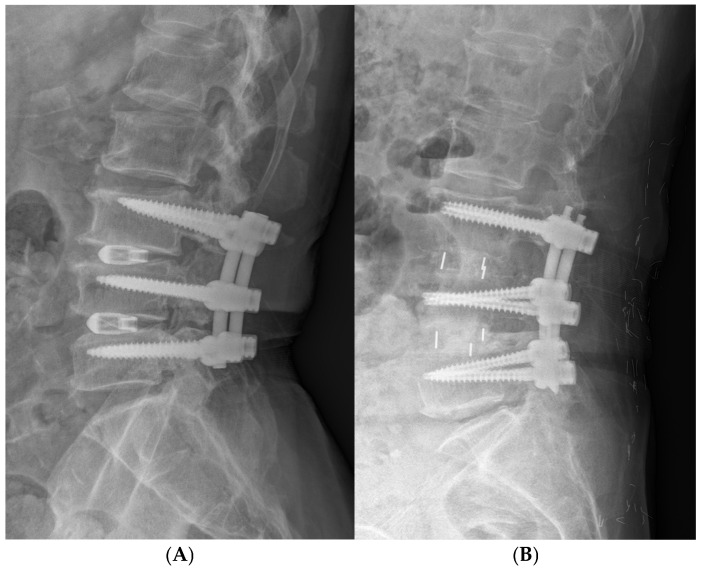
Standing lateral radiographs obtained at one-year postoperative follow-up after TLIF L3–4–5 (**A**) and LLIF L3–4–5 (**B**) and demonstrating maintained alignment and fusion status.

Surgical procedures were performed by two spine surgeons. All TLIF procedures were performed by H-J K, and All LLIF procedures were performed by S-M P. Both surgeons had at least 10 years of experience with their respective techniques (Figure 1).

For TLIF procedures, patients were positioned prone on a Jackson table. A midline posterior approach was utilized, with unilateral or bilateral facetectomy performed as dictated by the preoperative pathology. Complete discectomy was achieved, and the intervertebral space was prepared with sequential distraction and endplate preparation. A 3D printed titanium cage (CONDUIT PLIF; DePuy Synthes, Raynham, MA, USA) packed with locally harvested autograft supplemented with demineralized bone matrix was inserted. Segmental fixation was achieved using polyaxial pedicle screws and rods (Expedium system; DePuy Synthes, Raynham, MA, USA) that connected the screws on each side.

For LLIF procedures, patients were positioned in the right lateral decubitus position. A retroperitoneal approach was performed through a skin incision centred over the target disc space under fluoroscopic guidance. The discectomy was performed as extensively as possible with care taken to achieve excellent arthrodesis without damaging the endplates. Following discectomy, a carbon fibre reinforced PEEK cage (Cougar LS; DePuy Synthes, Raynham, MA, USA) filled with allograft bone was tamped into place with fluoroscopic guidance. All patients were repositioned prone for percutaneous posterior pedicle screw fixation (Viper system; DePuy Synthes, Raynham, MA, USA).

### 2.4. Outcome Measures

Baseline demographic and clinical data were collected preoperatively, including age, sex, height, body mass index (BMI), diagnosis, operative levels, diabetes and smoking history. Follow-up assessments were conducted at 3, 6, 12, and 24 months postoperatively. Radiographic measurements were performed by a single blinded observer, a spine surgeon with >4 years of experience who was not involved in patient treatment. Intra-observer reliability test was not conducted.

The primary outcome was the SLA at the operated level, measured on standing lateral lumbar radiographs. SLA was measured at each surgical level and subsequently compared between the TLIF and LLIF groups.

The secondary outcomes included disc height (DH) at the surgical level, lumbar lordotic angle (LLA), and sagittal vertical axis (SVA). The clinical outcomes comprised patient-reported outcome measures including pain intensity assessed using the visual analogue scale (VAS) for low back pain (VAS-LBP) and leg pain (VAS-LE), functional disability measured by the ODI, and health-related quality of life evaluated using the EuroQol-5 Dimension questionnaire (EQ-5D) index. All questionnaires were administered in validated Korean versions.

Surgical complications were systematically recorded and categorized. Major complications included dural tear, surgical site infection, deep venous thrombosis, epidural hematoma requiring revision, hardware failure, and new neurological deficit. Approach-related complications specific to LLIF, including sympathetic chain injury and psoas weakness, were also documented. Adjacent segment disease (ASD), defined as new symptomatic degeneration contiguous to the fused level, was recorded during follow-up.

### 2.5. Statistical Analysis

Comparisons between the two groups were performed using independent t-tests for normally distributed continuous variables and Mann–Whitney U tests for non-normally distributed continuous variables. The Shapiro–Wilk test was used to assess the normality of the data distribution. Chi-squared or Fisher’s exact tests were used for categorical variables.

Longitudinal analysis of repeated-measures outcomes was performed using linear mixed models (LMM). The models included fixed effects for group (LLIF vs. TLIF), time (as a categorical variable), and the group-by-time interaction, with random intercepts for individual subjects to account for within-subject correlation. This approach accommodates unbalanced designs and missing data under the missing-at-random assumption. For missing data, last observation carried forward or backward imputation was applied. It was applied only as a supplementary approach to preserve data completeness for descriptive and secondary analyses.

Statistical significance was defined as *p*-value < 0.05. All analyses were performed using R (version 4.1.0; R Foundation for Statistical Computing, Vienna, Austria).

### 2.6. Ethical Considerations

This study was conducted in accordance with the Declaration of Helsinki and approved by the Institutional Review Board of Seoul National University Bundang Hospital (IRB No. B-2007-627-308 and 12 August 2020). Written informed consent was obtained from all participants prior to enrollment.

## 3. Results

### 3.1. Patient Demographics and Baseline Characteristics

A total of 117 patients were enrolled in this study, with 17 patients in the LLIF group and 100 patients in the TLIF group. At the 24-month follow-up, 16 patients (94.1%) in the LLIF group and 93 patients (93.0%) in the TLIF group completed the study protocol. One patient in the LLIF group and seven patients in the TLIF group were lost to follow-up.

There were no significant differences in baseline demographic data between the two groups (Table 1). All patients had a primary diagnosis of lumbar spinal stenosis. Concurrent degenerative spondylolisthesis was present in 7 patients (41.2%) in the LLIF group and 27 patients (27.0%) in the TLIF group (*p* = 0.367). Single-level fusion was performed in 78.4% of LLIF patients and 80.3% of TLIF patients (*p* = 0.632). The most commonly operated level was L4-5, and no LLIF procedures were performed at the L5-S1 level. Prior lumbar surgery at non-index levels was reported in 23.5% of LLIF patients compared to 7.0% of TLIF patients (*p* = 0.054).

### 3.2. Radiographic Outcomes

Both groups demonstrated no significant differences in radiographic outcomes over the 24-month observation period (Table 2). The mean SLA at baseline was 17.47° ± 4.80° in the LLIF group and 16.97° ± 5.65° in the TLIF group (*p* = 0.706). Both groups showed increases in SLA over time, reaching 20.51° ± 4.82° and 20.15° ± 5.79° at 24 months in the LLIF and TLIF groups, respectively (*p* = 0.782). Linear mixed model analysis revealed no significant between-group effect (*p* = 0.165), within-subject time effect, or group-by-time interaction for SLA.

DH demonstrated improvement in both groups, but no significant between-group difference was observed (*p* = 0.518). LLA remained stable across all time points in both groups (*p* = 0.718), with mean values ranging from 38.68° to 44.80° throughout follow-up. SVA exhibited substantial variability, with a wide standard deviation range (SD range: 12–17 mm), indicating high individual differences in sagittal balance recovery, but remained comparable between groups at all follow-up (*p* = 0.866). Linear mixed model analysis revealed no significant within-subject (time) effect, suggesting that none of the parameters changed significantly over time (*p* > 0.05). The interaction effect (group × time) was also not significant, indicating that LLIF and TLIF followed a similar progression pattern.

### 3.3. Clinical Outcomes

Patient-reported outcomes demonstrated significant improvements in both groups, with some notable between-group differences (Table 3). Mean LBP decreased from 5.65 ± 3.18 at baseline to 2.82 ± 1.18 at 24 months in the LLIF group, and from 6.51 ± 2.55 to 4.48 ± 1.50 in the TLIF group. Baseline leg pain VAS scores were significantly higher in the TLIF group (7.43 ± 2.26) compared to the LLIF group (5.35 ± 3.45, *p* = 0.012). Leg pain scores decreased from 5.35 ± 3.45 to 2.82 ± 1.18 in the LLIF group and from 7.43 ± 2.26 to 4.66 ± 2.01 in the TLIF group over 24 months. Functional outcomes improved substantially in both groups. The ODI scores demonstrated a decrease from 23.65 ± 5.01 at baseline to 10.64 ± 4.32 at 24 months follow-up, while the TLIF group improved from 22.34 ± 7.95 to 14.71 ± 5.48. EQ-5D index scores improved from 0.34 ± 0.21 to 0.59 ± 0.18 in the LLIF group and from 0.33 ± 0.26 to 0.55 ± 0.22 in the TLIF group. The LLIF demonstrated significantly greater functional improvement compared to TLIF (*p* < 0.05). Both groups showed the greatest improvement at 3 months postoperatively (LLIF: 0.74 ± 0.15; TLIF: 0.51 ± 0.22), with subsequent modest decline. No significant difference was observed between groups (*p* = 0.091).

LMM analysis was performed to evaluate between-group effects, time effects, and group-by-time interactions for all clinical outcomes. For LBP, a significant time effect was observed (*p* = 0.038), but the between-group effect (*p* = 0.069) and interaction (*p* = 0.541) were not significant. Leg pain demonstrated both a significant between-group effect (*p* < 0.001) and time effect (*p* = 0.007), with the TLIF group showing consistently higher pain scores; the interaction was not significant (*p* = 0.429). The ODI showed a significant time effect (*p* = 0.002), but the between-group effect was not significant (*p* = 0.335) despite significant differences at individual time points on univariate analysis. The interaction was not significant (*p* = 0.656). For EQ-5D, both the time effect (*p* = 0.056) and between-group effect (*p* = 0.059) approached but did not reach significance, with no significant interaction (*p* = 0.754). While univariate comparisons at individual time points showed statistically significant differences between groups for several outcomes, LMM analysis revealed no significant difference in group × time effect.

### 3.4. Complications

The complication profiles differed between groups, reflecting approach-specific risks. Incidental durotomy occurred in 4 patients (4.0%) in the TLIF group and none in the LLIF group. All dural tears were repaired primarily without sequelae. Surgical site infection requiring debridement and antibiotic therapy was observed in 2 TLIF patients (2.0%). One patient (1.0%) in the TLIF group developed an epidural hematoma requiring evacuation on postoperative day 1, and one patient (1.0%) experienced hardware failure necessitating revision at 14 months. Sympathetic chain injury manifesting as ipsilateral lower extremity warmth occurred in 5 LLIF patients (29.4%), all of which resolved spontaneously within 3 months. Transient hip flexion weakness attributed to psoas muscle irritation was observed in 2 LLIF patients (11.8%), with complete resolution by 6 weeks postoperatively. These approach-specific complications were not observed in the TLIF group.

Wound dehiscence requiring secondary closure occurred in 3 TLIF patients (3.0%). Vertebral compression fractures at non-index levels were identified in 2 TLIF patients (2.0%) during follow-up. Adjacent segment disease requiring additional intervention developed in 2 patients in each group (LLIF: 11.8%; TLIF: 2.0%) by the 24-month follow-up. Urinary tract infections were documented in the perioperative period in the TLIF group only and no instances of deep venous thrombosis or new permanent neurological deficit were recorded in either group. Overall complications occurred in 14 of 100 patients (14.0%) in the TLIF group and in 6 of 17 patients (35.3%) in the LLIF group. The difference did not reach statistical significance (*p* = 0.074).

## 4. Discussion

In this study, we investigated the clinical, radiographic, and surgical outcomes of LLIF and TLIF techniques for lumbar fusion surgery. While radiographic assessments (SLA, DH, LLA, SVA) revealed no statistically significant differences between the two techniques, both LLIF and TLIF showed similar trends in pain reduction over time. These findings suggest that both techniques provide comparable spinal realignment, disc height restoration, and pain and functional improvement over the 24-month follow-up period.

Our results show that both groups achieved comparable radiologic restoration. Several recent meta-analyses have reported that LLIF has an advantage in SLA improvement compared to TLIF [9,10,11]. However, in this study, both groups demonstrated similar SLA correction. (+3.04° vs. +3.18°). This observation can be explained by several reasons. First, recent advancements in cage design for TLIF have significantly improved surgical outcomes. The introduction of lordotic designs and 3D-porous titanium cages has reduced subsidence rates, while hyperlordotic cages now available enable more effective restoration of SLA [12,13]. Second, Cage position can contribute to the degree of lordotic restoration achieved. Several previous studies have demonstrated that more anterior positioning of the cage can significantly influence the degree of SLA restoration in TLIF [14,15,16]. Lovecchio et al. reported no significant difference in SLA restoration and cage position between LLIF and TLIF, suggesting that surgical technique and cage positioning strategy may be more important than the choice of approach itself [16]. Both surgical procedures were performed by two different experienced surgeons, which may have introduced variability in cage positioning strategies. While both surgeons are spine specialists, individual surgical techniques could have influenced SLA restoration achievement. These findings suggest that meticulous surgical planning are more critical determinants of radiographic outcomes than approach selection alone [17,18].

The LMM analysis demonstrated comparable clinical outcomes between the two surgical techniques. The higher baseline leg pain in the TLIF group (7.43 vs. 5.35, *p* = 0.027) may be explained by potentially surgeon-specific patient selection and disease severity differences. TLIF may be preferred for severe central stenosis with facet joint hypertrophy requiring direct decompression. While leg pain has revealed a significant difference between-group effect (*p* < 0.001) favouring LLIF, the absence of group-by-time interaction (*p* = 0.429) indicates that both groups followed parallel recovery trajectories, merely offset by higher baseline leg pain in TLIF group (7.43 vs. 5.35, *p* = 0.027). Earlier studies have reported favourable clinical outcomes for LLIF over TLIF. Specifically, LLIF has been associated with reduced postoperative hospital days and lower VAS scores compared to TLIF [19]. The significant time effect for low back pain (*p* = 0.038) and ODI (*p* = 0.002) confirms the efficacy of both procedures. However, the between-group effect was not statistically significant, demonstrating no meaningful difference between the two groups. Although LMM analysis revealed no significant group-by-time effect, LLIF showing superior LBP VAS score at 3 months (1.71 vs. 3.85, *p* = 0.001) and at 24 months (2.82 vs. 4.48, *p* < 0.05) compared with TLIF. LLIF involves less disruption of the posterior paraspinal musculature, potentially facilitating more rapid early recovery and long-term pain relief. Pillastrini et al. demonstrated that exercise-based interventions can enhance multifidus muscle tropism, suggesting that such rehabilitation may contribute to the reduction in LBP in TLIF [20].

In terms of surgical complications, distinct patterns were observed between the two groups. TLIF was associated with a higher rate of major surgical complications, including dural tears (4 cases), surgical site infection (2 cases), and wound dehiscence (3 cases). These complications are likely due to the posterior approach’s direct manipulation of neural structures and soft tissue dissection. Conversely, LLIF had a greater incidence of complications related to the lateral approach, such as sympathetic chain injury (5 cases) and psoas weakness (2 cases), which are often attributed to retraction-related effects on the psoas muscle and neural structures [21]. Despite these differences, the overall complication rate did not show a statistically significant difference between the two groups (*p* = 0.074). In recent meta-analysis, there were no significant differences in surgical complications between LLIF and TLIF, which is consistent with our findings [19].

This study has several limitations. First, the LLIF group (n = 17) was significantly smaller than the TLIF group (n = 100), limiting statistical power and generalizability. Second, both surgical procedures were performed by two different surgeons, introducing potential bias. Surgeon-related performance bias, including technical proficiency and individual surgical technique, may have influenced outcomes beyond the inherent characteristics of each approach. Finally, important outcomes such as estimated blood loss, hospital day and fusion rate were not evaluated. Nonetheless, the prospective observational comparative study design lends substantial strength to the validity and clinical relevance of the findings. Future studies should incorporate randomized controlled designs and advanced statistical models to account for baseline differences and individual variability.

## 5. Conclusions

This study demonstrates that LLIF and TLIF have comparable overall safety profiles, with no significant differences in radiographic outcomes or pain reduction over a 24-month follow-up period. Both techniques are effective for lumbar fusion but require different surgical considerations to mitigate approach-specific complications. While the single-centre design and sample size imbalance limit generalizability, this prospective observational study provides clinically relevant data for the selection of each surgical technique.

## Figures and Tables

**Table 1:** Baseline Characteristics between two groups.

Variable	LLIF (n = 17)	TLIF (n = 100)	*p*-Value
Age (years)	62.4 (10.7)	63.8 (9.5)	0.421
Sex (M/F)	7/10	40/60	1.000
Height (cm)	161.3 (8.4)	162.5 (7.9)	0.312
BMI (kg/m^2^)	24.1 (3.6)	24.8 (3.4)	0.187
Diagnosis			
Spinal stenosis	17	100	1.000
Spondylolisthesis	7 (41.2%)	27 (27.0%)	0.367
Segments			0.103
L2-3	1	3	
L3-4	6	33	
L4-5	15	72	
L5-S1	0	30	
Single Level (%)	78.4	80.3	0.632
Double Level (%)	21.6	19.7	0.632
PMHx			
Diabetes	4 (23.5%)	11 (11.0%)	0.230
Smoking	3 (17.6%)	16 (16.0%)	1.000
Previous Surgery	4 (23.5%)	7 (7.0%)	0.054

Values are represented as Mean (SD) and n (%).

**Table 2:** Longitudinal comparison of radiographic outcomes between LLIF and TLIF groups.

Variable	Time Point	LLIF (n = 16)	TLIF (n = 93)	*p*-Value
SLA (degree)	Baseline	17.47 (4.80)	16.97 (5.65)	0.706
	3 Months	17.11 (4.66)	17.12 (5.97)	0.994
	6 Months	19.43 (3.30)	17.65 (5.36)	0.073
	12 Months	21.55 (4.00)	19.70 (5.55)	0.109
	24 Months	20.51 (4.82)	20.15 (5.79)	0.782
DH (mm)	Baseline	6.34 (2.28)	7.20 (3.03)	0.182
	3 Months	7.51 (3.18)	7.81 (3.06)	0.714
	6 Months	8.62 (3.65)	9.24 (3.07)	0.518
	12 Months	11.06 (3.26)	10.15 (2.91)	0.295
	24 Months	9.95 (2.21)	10.21 (2.89)	0.681
LLA (degree)	Baseline	39.62 (13.01)	40.56 (10.16)	0.780
	3 Months	43.90 (10.01)	38.68 (10.90)	0.062
	6 Months	38.91 (8.27)	41.34 (8.63)	0.278
	12 Months	44.24 (12.11)	44.56 (10.05)	0.920
	24 Months	44.80 (7.06)	44.21 (10.38)	0.768
SVA (mm)	Baseline	31.89 (17.08)	34.71 (14.63)	0.528
	3 Months	36.58 (13.88)	36.38 (13.86)	0.958
	6 Months	38.95 (14.00)	35.39 (17.76)	0.362
	12 Months	36.24 (12.51)	34.90 (16.20)	0.700
	24 Months	35.49 (16.94)	36.25 (13.31)	0.862

Values are represented as Mean (SD). SLA: Segmental lordotic angle, DH: Disc height, LLA: Lumbar lordotic angle, SVA: Sagittal vertical axis.

**Table 3:** Longitudinal comparison of clinical outcomes between LLIF and TLIF groups.

Variable	Time Point	LLIF (n = 16)	TLIF (n = 93)	*p*-Value
LBP (VAS)	Baseline	5.65 (3.18)	6.51 (2.55)	0.301
	3 Months	1.71 (2.02)	3.85 (2.68)	0.001
	6 Months	4.13 (2.53)	4.37 (2.21)	0.532
	12 Months	3.31 (1.88)	4.23 (2.14)	0.014
	24 Months	2.82 (1.18)	4.48 (1.50)	<0.05
Leg pain (VAS)	Baseline	5.35 (3.45)	7.43 (2.26)	0.027
	3 Months	1.47 (1.92)	4.14 (2.36)	<0.001
	6 Months	0.87 (1.25)	4.25 (2.22)	<0.001
	12 Months	2.54 (1.67)	4.08 (2.02)	<0.05
	24 Months	2.82 (1.18)	4.66 (2.01)	<0.05
ODI	Baseline	23.65 (5.01)	22.34 (7.95)	0.375
	3 Months	12.06 (4.32)	15.96 (5.67)	<0.05
	6 Months	12.53 (4.21)	15.47 (4.96)	0.032
	12 Months	10.92 (3.87)	14.78 (4.52)	0.028
	24 Months	10.64 (4.32)	14.71 (5.48)	<0.05
EQ-5D	Baseline	0.34 (0.21)	0.33 (0.26)	0.647
	3 Months	0.74 (0.15)	0.51 (0.22)	<0.001
	6 Months	0.66 (0.17)	0.53 (0.20)	<0.05
	12 Months	0.56 (0.18)	0.56 (0.19)	0.091
	24 Months	0.59 (0.18)	0.55 (0.22)	0.091

Values are represented as Mean (SD). VAS: Visual analogue scale, LBP: Low back pain, ODI: Oswestry Disability Index, EQ-5D: EuroQol-5 Dimension questionnaire.

## Data Availability

The data that support the findings of this study are available from the corresponding author upon reasonable request.

## Abbreviations

ASD	adjacent segment disease
BMI	body mass index
DH	disc height
EQ-5D	EuroQol-5 Dimension questionnaire
IRB	Institutional Review Board
LBP	low back pain
LIF	lumbar interbody fusion
LLA	lumbar lordotic angle
LLIF	lateral lumbar interbody fusion
LMM	linear mixed model
MCID	minimal clinically important difference
ODI	Oswestry Disability Index
SLA	segmental lordotic angle
SVA	sagittal vertical axis
TLIF	transforaminal lumbar interbody fusion
VAS	visual analogue scale.
